# Developing a targeted, theory-informed implementation intervention using two theoretical frameworks to address health professional and organisational factors: a case study to improve the management of mild traumatic brain injury in the emergency department

**DOI:** 10.1186/s13012-015-0264-7

**Published:** 2015-05-25

**Authors:** Emma J. Tavender, Marije Bosch, Russell L. Gruen, Sally E. Green, Susan Michie, Sue E. Brennan, Jill J. Francis, Jennie L. Ponsford, Jonathan C. Knott, Sue Meares, Tracy Smyth, Denise A. O’Connor

**Affiliations:** National Trauma Research Institute, The Alfred, Monash University, Melbourne, Australia; Department of Surgery, Central Clinical School, Monash University, Melbourne, Australia; Department of Trauma, The Alfred Hospital, Melbourne, Australia; School of Public Health and Preventative Medicine, Monash University, Melbourne, Australia; Department of Clinical, Educational and Health Psychology, University College London, London, UK; School of Health Sciences, City University London, London, UK; Monash-Epworth Rehabilitation Research Centre, Epworth Hospital, Melbourne, Australia; School of Psychological Sciences, Monash University, Melbourne, Australia; Melbourne Medical School, The University of Melbourne, Melbourne, Australia; Department of Emergency Medicine, Royal Melbourne Hospital, Melbourne, Australia; Department of Psychology, Macquarie University, Sydney, Australia; Emergency Department, Westmead Hospital, Westmead, Australia

**Keywords:** Intervention design, Intervention development, Theory use, Theoretical domains framework, Diffusion of innovations in service organisations

## Abstract

**Background:**

Despite the availability of evidence-based guidelines for the management of mild traumatic brain injury in the emergency department (ED), variations in practice exist. Interventions designed to implement recommended behaviours can reduce this variation. Using theory to inform intervention development is advocated; however, there is no consensus on how to select or apply theory. Integrative theoretical frameworks, based on syntheses of theories and theoretical constructs relevant to implementation, have the potential to assist in the intervention development process. This paper describes the process of applying two theoretical frameworks to investigate the factors influencing recommended behaviours and the choice of behaviour change techniques and modes of delivery for an implementation intervention.

**Methods:**

A stepped approach was followed: (i) identification of locally applicable and actionable evidence-based recommendations as targets for change, (ii) selection and use of two theoretical frameworks for identifying barriers to and enablers of change (Theoretical Domains Framework and Model of Diffusion of Innovations in Service Organisations) and (iii) identification and operationalisation of intervention components (behaviour change techniques and modes of delivery) to address the barriers and enhance the enablers, informed by theory, evidence and feasibility/acceptability considerations. We illustrate this process in relation to one recommendation, prospective assessment of post-traumatic amnesia (PTA) by ED staff using a validated tool.

**Results:**

Four recommendations for managing mild traumatic brain injury were targeted with the intervention. The intervention targeting the PTA recommendation consisted of 14 behaviour change techniques and addressed 6 theoretical domains and 5 organisational domains. The mode of delivery was informed by six Cochrane reviews. It was delivered via five intervention components : (i) local stakeholder meetings, (ii) identification of local opinion leader teams, (iii) a train-the-trainer workshop for appointed local opinion leaders, (iv) local training workshops for delivery by trained local opinion leaders and (v) provision of tools and materials to prompt recommended behaviours.

**Conclusions:**

Two theoretical frameworks were used in a complementary manner to inform intervention development in managing mild traumatic brain injury in the ED. The effectiveness and cost-effectiveness of the developed intervention is being evaluated in a cluster randomised trial, part of the Neurotrauma Evidence Translation (NET) program.

## Background

Guidance for developing complex interventions, such as those focussed on implementation, advocate the use of theory in the intervention development process [1]. It is argued that interventions are more likely to be effective if they target causal determinants of behaviour and behaviour change, and theory can be useful in gaining an understanding of these causal mechanisms [2]. In addition, there have been calls for better descriptions and reporting of implementation interventions to enable replication and refinement of interventions [3, 4]. Few studies report the rationale, process of development and detailed description of the intervention content, mode of delivery and the setting in which it is delivered to inform replication and/or refinement of interventions [5–7].

There are several approaches to the use of theory for developing interventions [2, 8–10], but there is currently no consensus on how best to select or apply theory. Multiple theories and theoretical frameworks of individual and organisational behaviour change exist, but choosing an appropriate theory can be challenging [9, 11–13]. Drawing on multiple relevant theories rather than a single theory is considered to facilitate a more comprehensive assessment of potential determinants of change and therefore an intervention that is more likely to be effective [9].

The Theoretical Domains Framework (TDF) [14, 15] is a comprehensive framework of 14 theoretical domains from 33 behaviour change theories and 128 constructs. It was developed using an expert consensus and validation process to identify an agreed set of theoretical domains that could be used when studying implementation and developing implementation interventions. The TDF has been successfully used in a wide range of settings, including the emergency department (ED) setting, to explore factors influencing clinical behaviour change and to design implementation interventions [16]. The ED environment is complex and has unique characteristics that can have an impact on its responsiveness to change, e.g. high staff turnover, lack of follow-up and a high number of decisions per unit of time [17].

Mild traumatic brain injury (mTBI) or concussion accounts for up to 90 % of patients who present to the ED with a traumatic brain injury (TBI) [18, 19] and has an incidence rate of between 100 and 300/100,000 inhabitants per year [20]. A recent study from the USA found that between the years 2006 and 2010, the rate of increase in TBI visits was eightfold greater than the rate of increase of total ED visits, and this increase was largely due to mTBI patients [21]. Mild TBI patients are predominately managed in the ED and discharged within hours [22]. While the majority will make a full recovery within a few weeks or months, approximately 15–25 % of patients will go on to have post-concussion symptoms, e.g. subjective, self-reported ongoing headaches and cognitive problems [23, 24]. A small minority (approximately 1 %) deteriorate and require neurosurgical intervention [25].

Evidence-based guideline recommendations are available to guide the care of patients with mTBI in the ED. However, studies indicate there is variability in management practices and care is often inconsistent with guideline recommendations [26–32]. The Neurotrauma Evidence Translation (NET) program is a 5-year knowledge translation program that aims to increase the uptake of research evidence to inform the care of patients who have sustained a TBI [33]. One of the program’s objectives is to systematically develop and evaluate a targeted, theory- and evidence-informed intervention to increase the uptake of evidence in the ED management of mTBI. The intervention will be implemented in EDs across the states of Australia and its effectiveness will be evaluated in a cluster randomised trial [34].

Previous implementation research undertaken in the ED setting has identified influential factors at the levels of the individual clinician, the environment and the organisation [35–37]. Although some organisational constructs are represented in the TDF (e.g. under the domains ‘Environmental Context and Resources’, ‘Social Influences’, ‘Social/Professional Role and Identity’ and ‘Behavioural Regulation’), further elaboration of the framework to include organisation-level influences has been suggested as a means of enhancing the usefulness of the framework [16]. Therefore, a conceptual model for considering potential factors influencing the organisational context of organisations was chosen to elaborate these domains. There are several frameworks available to explore the contextual factors influencing implementation of interventions in complex organisations such as the ED [38, 39]. Context can be defined as ‘influences which interact with each other, and interact with the implementation process’ [40]. The Model of Diffusion of Innovations in Service Organisations [41] was chosen as it was developed through a systematic review of the literature, covering 13 research areas in various disciplines (e.g. sociology, psychology, organisation and management), and the domains exploring organisational characteristics were comprehensive and deemed relevant for this setting. It identifies the main domains or areas in which factors influence the uptake and implementation of interventions in organisations. This model is only one way to investigate this issue but it is important to apply a model that has been developed from a strongly organisational perspective.

This paper describes the process of developing a targeted, theory- and evidence-informed intervention aiming to improve the management of mTBI in the ED, drawing on these two theoretical frameworks. It discusses the manner in which these frameworks were used in a complementary way to develop the intervention components and provides descriptions of the behaviour change techniques (BCTs) and modes of delivery used in the intervention and the causal processes targeted by the BCTs.

## Methods

**Fig. 1:**
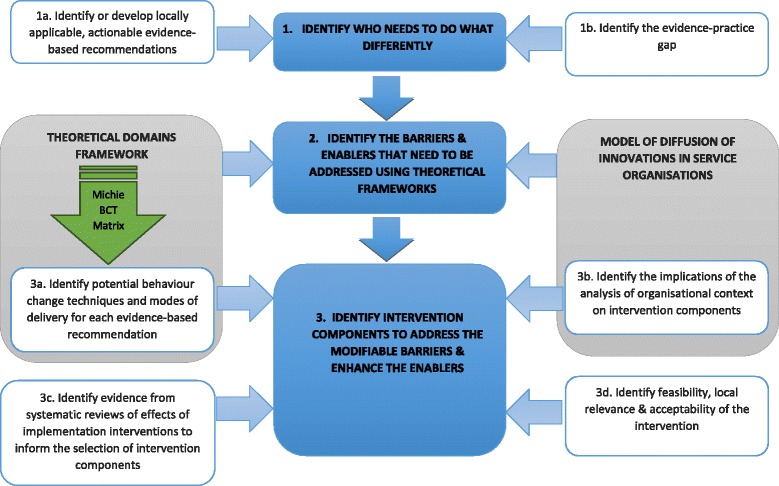
Process of developing a targeted, theory-informed intervention using two theoretical frameworks

A stepped approach was used to develop the intervention (see Fig. 1) and is described in detail below. This approach was developed drawing on the methods outlined by French et al. [9], which was used to design an intervention to improve the management of low back pain in general practice [42].

### Identify who needs to do what, differently

#### Identify or develop locally applicable, actionable evidence-based recommendations

In the absence of an up to date, locally relevant evidence-based guideline (EBG), a systematic search to identify guidelines relevant to the management of mTBI was undertaken and the quality of the identified EBGs was rated using the Appraisal of Guidelines Research and Evaluation (AGREE) Instrument [43]. Recommendations from guidelines that met our quality criteria were extracted from the EBGs and included in a recommendation matrix [32]. To determine the focus of our study, we identified strong evidence-based recommendations (i.e. grade A or B) in key clinical management areas (i.e. present in the majority of included EBGs). An additional search of the literature from the date of the last search of the most up to date EBG was undertaken to identify additional studies. Evidence overview tables were developed that incorporated the supporting evidence from the recommendation matrix and the additional studies. These tables were discussed at an international consensus meeting to agree upon the evidence statements. Eleven participants attended the meeting representing a range of organisations located in Australia, the USA and Canada including major trauma centres and/or foundations. All participants had a background in (clinical) research with all but three of the participants being clinically trained. Two local stakeholder meetings were then held in conjunction with relevant local clinical conferences in Melbourne, Australia to discuss the relevance of these evidence statements to the Australian ED setting, and to develop recommendations in the form of statements about who does what, when and how. The 1.5 h meetings were attended by 15 participants representing stakeholders in metropolitan and rural hospitals throughout Australia, in a variety of (clinical) roles [44].

#### Identify the evidence-practice gap

In order to quantify gaps between the recommendations agreed in ‘Identify or develop locally applicable, actionable evidence-based recommendations‘ section and current practice, two activities were undertaken: (i) a scoping search of the literature to identify studies conducted measuring practice patterns relevant to the management of mTBI patients in the ED, and (ii) a retrospective audit of the medical records of consecutive adult patients presenting with mTBI to the EDs of two inner-city hospitals in the Australian state of Victoria over a 2-month period (April to May 2011) [45].

### Identify the barriers and enablers that need to be addressed using theoretical frameworks

Semi-structured qualitative interviews were conducted with a sample of ED staff in the Australian state of Victoria to explore barriers and enablers to practice change [46]. Using a topic guide, questions relating to the TDF were used to investigate each of the recommended clinical behaviours [14] and questions relating to the Model of Diffusion of Innovations in Service Organisations were used to explore the organisational context in which the management of mTBI and change occurs [41]. Interviews were recorded and recordings were transcribed verbatim and anonymised. The interview transcripts were coded using thematic content analysis according to theoretical domains. Important (i.e. salient) domains were identified according to how frequently they were mentioned and/or deemed to be of high importance by the researchers or participant [47].

### Identify intervention components to address the modifiable barriers and enhance the enablers

Intervention components, that is, behaviour change techniques and modes of delivery, were identified as described below and in Fig. 1.

#### Identify potential behaviour change techniques and modes of delivery for each evidence-based recommendation

To select the behaviour change techniques (BCTs) most likely to bring about change for each recommended clinical behaviour, we mapped the important barriers and enablers, grouped by TDF domains (identified in ‘Identify the barriers and enablers that need to be addressed using theoretical frameworks‘ section), to appropriate BCTs using the matrix developed by Michie et al. [2]. The matrix links a taxonomy of BCTs to the theoretically derived theoretical domains that form the TDF and indicates which BCTs are likely to be effective in changing that particular domain. Additional techniques were identified from Cane et al. [48] that link BCTs from the BCT Taxonomy [49] to the refined TDF [15].

The BCTs were reviewed by the research team and potential modes of delivery were suggested. The BCTs and modes of delivery were reviewed in terms of feasibility and appropriateness for the local ED setting, informed by an analysis of the organisational context (see below).

#### Identify the implications of the analysis of organisational context on intervention components

Where factors derived from the analyses of organisational context were considered important and potentially modifiable, reviews/literature on specific theories and overviews of implementation interventions were consulted [41, 50–53] to identify intervention components that may be effective in targeting those factors. Other non-modifiable factors (moderators) were taken into consideration to maximise the likelihood that the intervention components were a good fit with the ED environment, e.g. influencing modes of delivery, duration of intervention components informing the choice between various BCTs. Implications of organisational context for intervention design were agreed in a research team meeting.

#### Identify evidence from systematic reviews of effects of implementation interventions to inform the selection of intervention components

Systematic reviews of interventions designed to improve healthcare systems and healthcare delivery published by the Cochrane Effective Practice and Organisation of Care (EPOC) Group [54] were searched in November 2012. Their findings, together with those from Grimshaw et al.’s overview of implementation interventions [55] were discussed in a research team meeting and intervention components were proposed. The overview provides a definition for each intervention, the likely mechanisms of action of interventions and comments on the practical delivery of interventions [55].

#### Identify feasibility, local relevance and acceptability of the intervention

Feasibility, local relevance and acceptability were assessed by the research team that included ED clinicians and behavioural scientists who used their experience to consider the practicality of delivery of the intervention components in the ED setting.

To facilitate reproducibility of the intervention, recommendations provided by the WIDER Group [3], TIDieR [4] and Proctor, et al. [6] were used to guide the development of descriptions of the intervention components. The following criteria were used to operationalise the intervention components: (1) characteristics of those delivering the intervention, (2) characteristics of the recipients (toward what or whom and at what level), (3) the setting (time and place of intervention), (4) intervention content, (5) mode of delivery, (6) intensity or dose (what frequency and intensity), (7) the duration (number of sessions, time) and (8) justification (theoretical, empirical or pragmatic).

## Results

### Identify who needs to do what, differently

#### Identify or develop locally applicable, actionable evidence-based recommendations

Six high-quality EBGs met the inclusion criteria and strong evidence-based recommendations were extracted. The quality of the EBGs and the extracted recommendations, along with the process of using these recommendations to develop locally applicable evidence-based recommendations, are described in detail elsewhere [32, 44]. Four target evidence-based recommendations were identified (see Table 1). To demonstrate the process of developing the intervention, the first of these recommendations will be used as an example throughout the paper: ‘post-traumatic amnesia (PTA) should be prospectively assessed in the ED using a validated tool’.

**Table 1:** Target evidence-based recommendations [32, 44, 46]

1.	Post-traumatic amnesia (PTA) should be prospectively assessed by nurses and/or doctors in the emergency department using a validated tool.
2.	Guideline-developed criteria or clinical decision rules should be used by doctors in the ED to determine the appropriate use and timing of CT imaging.
3.	Verbal and written information should be provided on discharge by nurses and/or doctors.
4.	Brief, routine follow-up consisting of advice, education and reassurance should be provided by General Practitioners (GPs), staff in the ED or rehabilitation clinicians.

#### Identify the evidence-practice gap

The scoping search of the literature identified studies from the UK, Ireland, USA, Canada and Norway that provided evidence of inter- and intra-hospital variability in the management of mTBI in the ED and the recommended clinical behaviours [26–31]. There were no published studies identified that reported rates of PTA assessment for mTBI.

The medical files of 206 consecutive patients presenting with mTBI at two EDs in the Australian state of Victoria were audited [45]. For the recommended behaviour, prospectively assessing patients for PTA using a validated tool, the rates of assessment of PTA in adults with mTBI were 0 % (95 % CI 0 to 14 %, *n* = 24) in one hospital and 31 % (95 % CI 24 to 39 %, *n* = 164) for the second [34, 45].

### Identify the barriers and enablers that need to be addressed using theoretical frameworks

Interviews with 42 ED staff from 13 hospitals were conducted between November 2010 and May 2011. The detailed findings from the interviews are described separately [46]. The key barriers and enablers for prospectively assessing patients for PTA using a validated tool were associated with six of the TDF domains ‘Knowledge’, ‘Environmental context and resources’, ‘Skills’, ‘Beliefs about consequences’, ‘Social/professional role and identity’ and ‘Beliefs about capabilities’ (see Table 2). Key organisational factors in relation to the management of this patient group, organising change in general and the organisational context in which the four recommended clinical behaviours take place are presented in Table 3.

**Table 2:** Key barriers and enablers for prospectively assessing post-traumatic amnesia using a validated tool [46]

TDF Domains	Themes
Knowledge	Limited knowledge of what PTA is, how to assess it and what tools are available to assess PTA in the ED.
Environmental context and resources	Mandated validated tool to assess PTA in the ED is not available in the ED. No space in the patient notes to include PTA information. ED has large workload and staff has increasing pressure to discharge patients quickly to free up beds.
Skills	Limited skills and training on how to assess PTA using validated tools.
Beliefs about consequences	Senior doctors do not see the additional benefits of using a validated tool to assess PTA, comfortable using their clinical experience. Using a tool to assess PTA is perceived as being more time consuming than using clinical questions and experience.
Social/professional role and identity	Assessing for PTA is seen as outside the role of the ED. Unsure of who is responsible for completing and promoting use of the validated PTA tool.
Beliefs about capabilities	Some ED clinicians find amnesia assessment difficult and there is inconsistency in assessment. Junior doctors find it more difficult due to their limited clinical experience. Nurses would prefer a more objective measure of amnesia and are open to the use of a validated tool.

**Table 3:** Key organisational factors and implications for the design and delivery of the intervention

Domains	Factors	Implications for intervention components
The intervention	Guideline-based intervention low compatibility with medical culture; good compatibility with nursing culture	Suggest nurses have the “main” lead role; suggest more training tasks to be done by nurses as well as use of actual tool
	Potential for reinvention needed (e.g. to reflect available resources)	Specify minimum local training; local opinion leaders determine how, by whom and when training is delivered. Communicate 3 recommended practices; EDs decide whether a pathway/protocol is developed from recommendations
	Changes need to be observable to keep momentum/commitment	Audit and feedback component [note: considered not feasible]
	Needs clear, unambiguous advantage over current practice	Communicate the evidence underpinning recommendations and health consequences
	High complexity of cross-unit change	Communicate 3 recommended practices; EDs determine how to integrated practice with care processes/pathways
System readiness for innovation	Relatively low tension for change/perceptions of collective change commitment for “acute part of management” (generally not perceived as in need of change)	Present baseline figures [note: considered not feasible]. Stress health impact for patients post discharge
	Mixed tension for change for management of longer-term symptoms (higher change commitment, but relatively low change efficacy)	Select different messages for different audiences
	Management driven agenda perceived to be very time-focused and not necessarily focused on high quality management from patient perspective	Communicate to senior leaders in stakeholder meeting the fact that the tool is very quick and may lead to shorter stay for patients in the ED
Implementation processes (change management practices)	Influence within social networks, not across (particularly in medical professions)	Identify multidisciplinary local opinion leader team (medical and nursing). Provide directors with a description of the types and characteristics of people suited to the role)
	Different professions have own systems in place for organising and communicating changes	Local opinion leaders determine the best way to communicate to staff
	Visible multidisciplinary leadership, use of ‘stable forces’	Include in local opinion leader training information about being ‘the constant reminder’ and the importance of leading by example
	Respected (informal) leaders	Provide ED Director with a description of characteristics of informal leaders
System antecedents for innovation	High turnover rates generally perceived to hamper implementation due to constant loss of tacit knowledge	Local opinion leaders deliver training and ensure training is provided to staff on different shifts. Provide ‘back-up’ materials (e.g. presentations with script) that local opinion leaders can distribute to staff unable to attend face-to-face training. Encourage local opinion leaders to integrate training and tools into work processes (e.g. materials for new staff). Involve stable workforce (consultants and nurses). Design brief training sessions that can be repeated regularly
	Little organisational slack, stretched environment	Provide EDs with reimbursement and communicate this in recruitment materials
	ED perceived to be open to change in general, positive culture in relation to change (relatively positive history of change)	Non-modifiable factor—included in process evaluation
	Stretched and hectic ED environment not conducive to learning and reflection	Design brief training sessions that can be fitted in easily and repeated often
	Constantly changing team-structure brings challenges to team-based learning	Include training on learning across professions in Train-the-Trainer day [note: unlikely to be feasible for local sessions]
	Lack of routine monitoring and feedback (as well as systems to support this); predominately reactive approaches to problem solving	Non-modifiable factor—included in process evaluation
	Coordination between various quality systems still very manual	Non-modifiable factor
Outer context	Being subspecialty at the entry-point of the hospital means many specialties have requests with respect to the management if they were to admit patients under their care	Organise stakeholder meetings and encourage discussions with stakeholders in the hospital
		Raise topic again later in project when thinking about sustaining the changes
	Absence of agreed cross-unit pathways/protocols	Encourage early discussions with range of stakeholders to maximise chances of sustaining the changes
	Agreement between different specialties generally difficult to organise	Encourage early discussions with range of stakeholders to maximise chances of sustaining the changes
	Accountability metrics very finance driven	Non-modifiable factor
	Financial systems focus on local costs; no entire patient care journey through the system; perceived absence of follow-up facilities	Communicate 3 recommended practices; EDs determine how to integrate practice with the care processes/pathways

### Identify intervention components to address the modifiable barriers and enhance the enablers

#### Identify potential behaviour change techniques and modes of delivery for each evidence-based recommendation

**Fig. 2:**
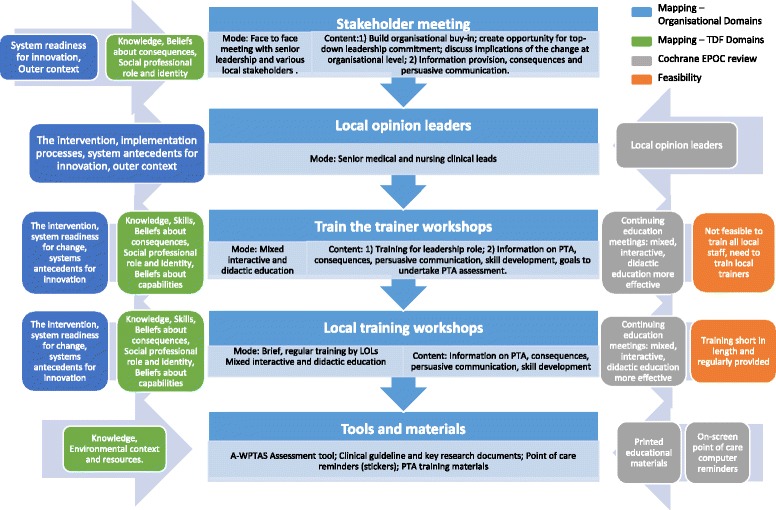
Intervention components to improve the recommended practice—post-traumatic amnesia should be prospectively assessed by clinical staff in the emergency department using a validated tool

Fourteen BCTs were selected to target the modifiable barriers and enhance the enablers for assessing PTA using a validated tool (grouped into six of the TDF domains). Table 4 provides details of the mapping process for selecting BCTs and the subsequent intervention components. For example, for the domain ‘Knowledge’, the BCTs ‘Information regarding behaviour, outcome’, ‘Antecedents’, ‘Health consequences’ and ‘Feedback on behaviour’ were advocated. Of the intervention components suggested, the provision of ‘Feedback on behaviour’ using audit data was not deemed feasible (see ‘Identify feasibility, local relevance and acceptability of the intervention‘ section). A summary of the intervention components that were decided upon for the PTA behaviour is included in Table 5 and illustrated in Fig. 2.

**Table 4:** Mapping of important barriers and enablers (grouped by TDF domains) for prospectively measuring post-traumatic amnesia using the Abbreviated-Westmead tool to behaviour change techniques and intervention components

TDF domains	BCTs advocated by Theory-Technique Matrix (including definitions) [2]	Additional BCTs (including definitions) suggested in Cane et al. [48]	Desirable intervention components	Proposed intervention components (including notes to justify omission of intervention components)
Knowledge	1. *Information regarding behaviour*, *outcome*	2. *Antecedents*	1. Information and training/education on what PTA is, the importance of assessing PTA in the ED, i.e. provide information on outcome and how to use the A-WPTAS tool	1,3. Information and training/education on what PTA is and how to use the A-WPTAS tool. Information on the importance and consequences of performing a PTA assessment
		3. *Health consequences*	2. Information on environmental situations, events that predict performance of the behaviour (i.e. when PTA is and is not measured)	2. Information on environmental situations, events that predict performance of the behaviour
		4. Feedback on behaviour	3. Include in (1)—consequences of performing behaviour	NOTES
			4. Incorporate in education feedback on the EDs performance (how many patients are assessed for PTA—informed by audit	4. Not feasible to undertake audit.
Environmental context and resources	1. *Environmental changes* (*e.g. object to facilitate behaviour*)	2. Restructuring the physical environment	1. Make available A-WPTAS tool and clinical pathway to staff—Intranet and hard copy	1. Make available A-WPTAS tool and clinical pathway to staff—Intranet and hard copy. Incorporation of PTA training materials in staff initiation materials, on the Intranet
				NOTES
		3. Restructuring the social environment	2. Change patient medical records to include amnesia recording	2. Not feasible to change patient medical records to include amnesia assessment (forms committee can take over a year)
		4. Prompts/cues	3. Reduce workload by increasing number of ED staff	3. Not feasible to increase staffing to reduce workload
			4. Prompts in the system/clinical pathway to undertake PTA assessment on all mTBI patients	4. Not feasible to include prompts in the system/clinical pathway to undertake PTA assessment on all mTBI patients
Skills	1. *Goal*/*target specified*: *behaviour or outcome*	None relevant.	1. Set goals to undertake PTA assessments on all mTBI patients	1. Set goals to undertake PTA assessments on all mTBI patients
	2. Monitoring		2–4. Monitoring (auditing) of behaviour and feedback to staff, e.g. review of patient records for number who have had an A-WPTAS assessment completed and how many were completed correctly	5–7. Training course including: skill development (how to do an A-WPTAS), modelling/demonstration by nurses, graded tasks (including scenarios ranging from simple to more complex), behavioural rehearsal with participants role playing, problem solving (how this will work in their hospital, how will they deal with pressures from doctors/wards)
	3. Self monitoring			
	4. Rewards; incentives (inc self evaluation)			
				NOTES
	5. *Graded task*, *starting with easy tasks*		5–7. Training course including: skill development (how to do an A-WPTAS), modelling/demonstration by nurses, graded tasks (including scenarios ranging from simple to more complex), behavioural rehearsal with participants role playing, problem solving (how this will work in their hospital, how will they deal with pressures from doctors/wards)	2–4. Audit data may be difficult to attain depending on the local patient record system in use. The level of details may be site specific
	6. *Increasing skills*: *problem solving*, *decision making*, *goal setting*			
	7. *Rehearsal of relevant skills*			
	8. *Modelling*/*demonstration of behaviour by others*			
Beliefs about consequences	1. Self monitoring	5. Emotional consequences	1. Monitoring (auditing) of behaviour and outcomes, e.g. review of patient records for number who have had an A-WPTAS assessment completed and how many were completed correctly	2. Persuasive communication from credible sources/opinion leaders to reinforce the benefits of performing a PTA assessment using the A-WPTAS
	2. *Persuasive communication*	6. Threat	2. Persuasive communication from credible sources/opinion leaders to reinforce the benefits of performing a PTA assessment using the A-WPTAS	3. Information/education on the importance of assessing of PTA in the ED and how to use the A-WPTAS tool
	3. *Information regarding behaviour*, *outcome*	7. *Pros and Cons*	3. Information/education on the importance of assessing of PTA in the ED and how to use the A-WPTAS tool	7. Include pros and cons of undertaking PTA assessment in training, persuasive messages
	4. Feedback	8. *Vicarious reinforcement*	4. Feedback to the nurses on performance, e.g. monitoring data and ways to improve	8. Include reinforcement messages from staff who are already using PTA
		9. Comparative imagining of future	7. Include pros and cons of undertaking PTA assessment in training, persuasive messages	13. Provide information on the consequences on the ED environment by undertaking PTA assessment—reducing discharge time. Include in education the benefits of undertaking an assessment of PTA using the A-WPTAS to patient flow, appropriateness of discharge and time (realistically) it takes to undertake one
				NOTES
		10. Outcomes	8. Include reinforcement messages from staff who are already using PTA	1. Audit data may be difficult to attain depending on the local patient record system in use. The level of details may be site specific
		11. Covert sensitisation	13. Provide information on the consequences on the ED environment by undertaking PTA assessment—reducing discharge time. Include in education the benefits of undertaking an assessment of PTA using the A-WPTAS to patient flow, appropriateness of discharge and time (realistically) it takes to undertake one	4. Without audit data it will be difficult to provide staff with feedback
		12. Covert conditioning	5, 6, 9, 10, 11, 12, 14—not relevant	
		13. *Social and environmental consequences*		
		14. Anticipated regret		
		15. *Salience of consequences*		
Social professional role and identity	1. *Social processes of encouragement*, *pressure*, *support*	No additional techniques listed in paper	1. Include persuasive messages from senior nurses/ED Director to convince that an A-WPTAS assessment is needed and it is part of their role	1. Include persuasive messages from senior nurses/ED Director to convince that an A-WPTAS assessment is needed and it is part of their role
Beliefs about capabilities	1. Self monitoring	10. Verbal persuasion to boost self efficacy	1. Monitoring (auditing) of behaviour, e.g. review of patient records for number who have had an A-WPTAS assessment completed, how many were completed correctly and number discharged in PTA	2,3,4. Training course including: skill development (what PTA is, how to incorporate A-WPTAS findings in discharge decision making), modelling, demonstration by doctors, graded tasks, rehearsal/role play with actors, problem solving (how this will work in their hospital, how will they deal with pressures from wards). Include difficult situations and ways to cope with these
	2. *Graded task*, *starting with easy tasks*	11. *Focus on past success*	2,3,4. Training course including: skill development (what PTA is, how to incorporate A-WPTAS findings in discharge decision making), modelling, demonstration by doctors, graded tasks, rehearsal/role play with actors, problem solving (how this will work in their hospital, how will they deal with pressures from wards)	5. Include persuasive messages from senior doctors/ED Director to convince that an A-WPTAS assessment is needed rather than just using clinical experience
	3. *Increasing skills*: *problem solving*, *decision making*, *goal setting*		Include difficult situations and ways to cope with these	11. Include in training the importance of focusing on previous successes
				NOTES
	4. *Rehearsal of relevant skills*		5. Include persuasive messages from senior doctors/ED Director to convince that an A-WPTAS assessment is needed rather than just using clinical experience	1. Audit data may be difficult to attain depending on the local patient record system in use. The level of details may be site specific
	5. *Social processes of encouragement*, *pressure*, *support*		6. Feedback to the nurses on performance, e.g. monitoring data and ways to improve	6. Without audit data it will be difficult to provide staff with feedback
	6. Feedback		11. Include in training the importance of focusing on previous successes	
	7. Coping skills		7,8,9,10—not relevant	
	8. Self talk			
	9. Motivational interviewing			

*BCTs* in italics are those deemed by the research team as particularly relevant for this particular behaviour

**Table 5:** Summary of intervention components to improve the prospective assessment of PTA using a validated tool

Key TDF domains	Proposed BCTs	Intervention components including the proposed BCTs
Knowledge	Information regarding behaviour, outcome	Training and education including: information on what PTA is and how to use a validated tool (abbreviated Westmead Post-traumatic Amnesia Scale- A-WPTAS) consequences of performing and not performing this behaviour, e.g. the benefits of undertaking an assessment of PTA using the A-WPTAS to patient flow, appropriateness of discharge and time (realistically) it takes to undertake one
	Antecedents	Information on environmental situations, events that predict performance of the behaviour (i.e. when PTA is not measured)
	Health consequences	
Environmental context and resources	Environmental changes	Resources
		Make available A-WPTAS tool and clinical pathway to staff—Intranet and hard copy. Incorporation of PTA training materials in staff initiation materials, on the Intranet
Skills	Goal/target specified behaviour or outcome	Training and education including: skill development (how to do an A-WPTAS), modelling/demonstration by nurses, graded tasks (including scenarios ranging from simple to more complex), behavioural rehearsal with participants role playing, problem solving (how this will work in their hospital, how will they deal with pressures from doctors/wards)
	Graded task, starting with easy tasks	Set goals to undertake PTA assessments on all mTBI patients and discuss ways of achieving this
	Increasing skills: problem solving, decision making, goal setting	
	Rehearsal of relevant skills	
	Modelling/demonstration of behaviour of others	
Beliefs about consequences	Persuasive communication	Training and education including: persuasive communication from credible sources/opinion leaders (senior nurses/ED Director) to reinforce the benefits of performing a PTA assessment using the A-WPTAS
	Social processes of encouragement, pressure, support	
	Pros and Cons	Include reinforcement messages from ED staff that are already using PTA
	Vicarious reinforcement	Information/education on the importance of assessing of PTA in the ED and how to use the A-WPTAS tool
	Social and environmental consequences	Include pros and cons of undertaking PTA assessment in training, persuasive messages
	Salience of consequences	Include reinforcement messages from staff who are already using PTA
		Provide information on the consequences on the ED environment by undertaking PTA assessment—reducing discharge time. Include in education the benefits of undertaking an assessment of PTA using the A-WPTAS to patient flow, appropriateness of discharge and time (realistically) it takes to undertake one. Include memorable consequences, e.g. patient examples
Social professional role and identity	Social processes of encouragement, pressure, support	Training and education including: persuasive messages from senior nurses/ED Director to convince that an A-WPTAS assessment is needed and it is part of their role
Beliefs about capabilities	Graded task, starting with easy tasks	Training and education including: emphasise the importance of focusing on previous successes [all other BCTs included in elements above]
	Increasing skills: problem solving, decision making, goal setting	
	Rehearsal of relevant skills	
	Social processes of encouragement, pressure, support	
	Focus on past success

#### Identify the implications of the analysis of organisational context on intervention components

Table 3 describes the implications of taking into account important factors from the analysis of the organisational context. Some overarching intervention components such as the stakeholder meeting and recruitment of local opinion leaders to deliver local training and the provision of reimbursement were proposed to overcome important organisational barriers and enhance enablers. These components were designed to address factors relevant to more than one clinical behaviour and, more broadly, to increase the compatibility of the intervention with the organisational setting. For instance, the primary reason for selecting local stakeholder meetings was to enhance organisational buy-in, e.g. provide the ED senior leadership with an opportunity to express commitment; to start the conversation with local stakeholders such as neuropsychologists and/or occupational therapists (as changes in ED practice may influence others in the hospital); to discuss how the recommended clinical behaviours fit with their current practices (e.g. protocols or pathways as relevant) and whether they foresaw any potential hurdles in introducing the intervention from an organisational point of view. The stakeholder meeting was also a first opportunity to introduce some of the BCTs selected to address TDF factors in relation to each recommended clinical behaviour (e.g. persuasive messages). Other organisational factors influenced decisions regarding the mode of delivery or feasibility of decisions (e.g. the high staff turnover rate in combination with an environment that is stretched means that local sessions need to be very brief, so they can be delivered frequently, and back-up materials (e.g. presentations with spoken script) need to be available for (new) staff to watch outside scheduled training moments. Fig. 2 illustrates how the organisational factors influenced the selection of intervention components targeting the assessment of PTA.

#### Identify evidence from systematic reviews of effects of implementation interventions to inform the selection of intervention components

Six Cochrane EPOC reviews were identified that focused on interventions to change practitioner behaviour and contained interventions deemed to be effective [56–61]. Table 6 includes the key findings from the reviews, the interventions’ hypothesised mechanisms of action, the practicalities of implementing them and the intervention components that were proposed by the research team when considering the findings of the reviews in relation to this implementation problem.

**Table 6:** Evidence from Cochrane EPOC reviews to inform intervention components

Cochrane review topic	Definition	Mechanism of action and practicality [71]	Key findings	Effect sizes	Proposed implications for intervention components
Continuing education meetings and workshops [56]	Participation of healthcare providers in conferences, lectures, workshops or traineeships	Didactic meetings target knowledge at the individual healthcare professional/peer group level. Interactive workshops target knowledge, attitudes and skills. Practicalities: commonly used with the main cost related to the release time for healthcare professionals and feasible in most settings.	Educational meetings alone or combined with other interventions can improve professional practice and the patient healthcare outcomes. The effect on professional practice tended to be small and varied between studies, and the effect on patient outcomes was generally less. It is not possible to explain the observed differences in effect with confidence but it appeared that higher attendance at the meetings was associated with greater effects, that mixed interactive and didactic education was more effective than either alone, and that the effects were less for more complex behaviours and less serious outcomes.	81 randomised controlled trials (11,000+ health professionals). Median absolute improvement in care of 6.0 % (IQR +1.8 % to +15.3 %).	Mixed interactive workshops and didactic education. [Note: may have smaller effects as mTBI is seen as a ‘less serious’ condition].
Local opinion leaders [57]	Use of providers nominated by their colleagues as ‘educationally influential’	Target: knowledge, attitudes and social norms of their peer group. Dependent on the existence of intact social networks within professional communities. Practicalities: resources required include cost of the identification method, training of opinion leaders and additional service costs.	Opinion leaders alone or in combination with other interventions may successfully promote evidence-based practice, but effectiveness varies both within and between studies. These results are based on heterogeneous studies differing in terms of type of intervention, setting, and outcomes measured. In most of the studies, the role of the opinion leader was not clearly described, and it is therefore not possible to say what the best way is to optimise the effectiveness of opinion leaders.	18 randomised controlled trials (296 hospitals and 318 primary care physicians). Median absolute improvement in care of 12 % (IQR +6.0 % to 14.5 %).	Local opinion leaders (clinical champions) to be nominated at each site and their characteristics and role to be clearly described.
Printed educational materials [58]	Distribution of published or printed recommendations for clinical care including clinical practice guidelines, audio-visual materials and electronic publications. The materials may have been delivered personally or through mass mailings.	Target: knowledge and potential skill gaps of individual healthcare professionals. Can be used to target motivation when written as a ‘persuasive communication’ but little evidence of being used in this way. Practicalities: commonly used and relatively low cost and feasible in most settings.	Printed educational materials when used alone and compared to no intervention may have a small beneficial effect on professional practice outcomes. There is insufficient information to reliably estimate the effect of PEMs on patient outcomes, and clinical significance of the observed effect sizes is not known. The effectiveness of PEMs compared to other interventions, or of PEMs as part of a multifaceted intervention, is uncertain.	14 randomised controlled trials and 31 interrupted time series studies (ITS). Median absolute risk difference in categorical practice outcomes was 0.02 when PEMs were compared to no intervention (range from 0 to +0.11).	Clinical guideline and key research publications to be provided.
Audit and feedback [59]	Any summary of clinical performance of healthcare over a specified period of time to change health professional behaviour as indexed by objectively measured professional practice in a healthcare setting or healthcare outcomes.	Target: ‘healthcare provider/peer groups’ perceptions of current performance levels and useful to create cognitive dissonance within healthcare professionals as a stimulus of behaviour change’. Practicalities: resources required to deliver audit and feedback including data extraction, analysis and dissemination costs. Feasibility dependent on availability of meaningful routine administrative data for feedback.	Audit and feedback generally leads to small but potentially important improvements in professional practice. The effectiveness of audit and feedback seems to depend on baseline performance and how the feedback is provided. Audit and feedback may be most effective when: (1) the health professionals are not performing well to start out with, (2) the person responsible for the audit and feedback is a supervisor or colleague, (3) it is provided more than once, (4) it is given both verbally and in writing and (5) it includes clear targets and an action plan.	140 randomised controlled trials. Median adjusted RD was 4.3 % (IQR 0.5 % to 16 %).	Regular audit and feedback provided by senior work colleague, provided in verbal and written format. Clear targets and action plan provided. [Note: Not feasible as ED rarely has routine administrative data for the behaviours targeted in this intervention.]
On-screen point of care computer reminders [60]	Patient or encounter specific information, provided verbally, on paper or on a computer screen, which is designed or intended to prompt a health professional to recall information.	Target: prompt health professionals to remember to do important things during patient interaction. Practicalities: resources necessary vary across the delivery mechanism.	Point of care computer reminders generally achieve small to modest improvements in provider behaviour. A minority of interventions showed larger effects, but no specific reminder or contextual features were significantly associated with effect magnitude. Further research must identify design features and contextual factors consistently associated with larger improvements in provider behaviour if computer reminders are to succeed on more than a trial and error basis.	28 randomised controlled trials. Median absolute improvement of care (process adherence) was 4.2 % (IQR +0.8 % to +18.8 %).	Encourage the use of point of care reminders, ideally computer reminders but if not feasible paper reminders such as sticker checklists on patient notes.
Educational outreach visits [61]	Use of a trained person who meets with providers in their practice settings to give information with the intent of changing the providers’ practice. The information given may have included feedback on the performance of the provider(s).	Target: an individual’s knowledge and attitudes (predominately target prescribing behaviours). Practicalities: considerable resources including the costs of detailers and preparation of materials.	Educational outreach visits alone or when combined with other interventions have effects on prescribing that are relatively consistent and small, but potentially important. Their effects on other types of professional performance vary from small to modest improvements, and it is not possible from this review to explain that variation.	69 randomised controlled trials involving 15,000 + health professionals. Median adjusted risk difference (RD) in compliance with desired practice was 5.6 % (IQR 3.0 % to 9.0 %). The adjusted RDs were highly consistent for prescribing (median 4.8 %, IQR 3.0 % to 6.5 % for 17 comparisons), but varied for other types of professional performance (median 6.0 %, IQR 3.6 % to 16.0 % for 17 comparisons). EOVs appeared to be slightly superior to audit and feedback.	[Note: Although it was found that EOVs were effective, its use in improving prescribing practice was deemed the most consistent result. As prescribing is not included in the target behaviours, its applicability was questioned. The considerable cost of including this component in an intervention that will be implemented in a large number of hospitals, located in diverse locations was also seen as a reason for not including it as an intervention component.]

*IQR* interquartile range

#### Identify feasibility, local relevance and acceptability of the intervention

The feasibility of delivering each of the proposed intervention components within the context of the ED was discussed by the research team, e.g. providing training and education in the ED with a high turnover of staff. The discussions resulted in the identification of five intervention components: local stakeholder meetings, identification of local opinion leader team (one medical and one nurse in each site), a train-the-trainer workshop for identified local opinion leaders, local training workshops facilitated by the trained local opinion leaders and the provision of tools and materials to prompt recommended behaviours. Several intervention components were deemed not feasible for implementation in the ED setting due to the limited time and resources available. These included changes to the electronic patient record system whereby a patient cannot be discharged without a patient information leaflet being printed out and the provision of regular audit and feedback data to clinical staff. Although there is evidence that regular audit and feedback can lead to improvements in professional performance [59], the outcomes of interest (including the primary outcome for the cRCT) are not routinely collected, and it was not feasible to deliver across the 34 included EDs. Table 7 provides details of the intervention components and how they were operationalised. Fig. 2 shows how the two frameworks influenced the design of the intervention for the recommended PTA behaviour, including details of where each of the intervention components originated and provides a justification for its inclusion, e.g. as part of the mapping process or evidence from EPOC reviews. The figure also includes overarching components and content (i.e. that apply not just to PTA, such as the stakeholder meetings and opinion leaders, which were identified as important to ensure an intervention that was suited to the organisational setting, rather than just targeting individual clinicians with behaviour-specific techniques).

**Table 7:** Operationalisation of intervention components

	Stakeholder meeting	Local opinion leader	Train the trainer	Local training workshops	Tools and materials to prompt recommended behaviours
Rationale for intervention component	Findings from interviews: Organisational and TDF factors	Findings from interviews: Organisational factors Cochrane EPOC reviews	Findings from interviews: Organisational and TDF factors Cochrane EPOC reviews feasibility information	Findings from interviews: Organisational and TDF factors Cochrane EPOC reviews feasibility information	Findings from interviews: Organisational and TDF factors Cochrane EPOC reviews feasibility information
Intervention content	Provide an opportunity to create buy-in at an organisational level and for senior leadership to express support. Provide opportunity to start conversation with stakeholders within hospital (outside ED) Key recommended behaviours and supporting evidence	Recruitment of local opinion leaders (one senior nurse and one medical lead from each participating hospital) to lead the project and train staff	Training and education including information/education on the key recommended practices and consequences of performing and not performing the behaviours, persuasive messages, skill development, modelling/demonstration and planning/implementation	Information/education on the key recommended practices and consequences of performing and not performing the behaviours, persuasive messages, skill development, modelling/demonstration	PTA assessment tool. Evidence-based discharge information sheet in different languages CT clinical decision tools lanyards. Checklist reminder stickers for patient records
	Endorsement letters from relevant ED colleges. Practicalities of how these will be implemented including discussion of local pathways and protocols and how to overcome anticipated barriers to implementation		Leadership and change management training (e.g. information on the importance and content of the role of the clinical leads)		Posters providing information on the evidence-based approach to managing patients with mTBI
Characteristics of those delivering the intervention	Senior research team clinicians	Not applicable	Senior research team clinicians	Local opinion leaders (nurse and medical)	Research team
			Clinical opinion leaders		
Characteristics of the recipient(s)	Local stakeholders (both clinical as well as change management, e.g. ED Director, nominated local opinion leaders and other stakeholders such as occupational therapists or radiologists)	Not applicable	Local opinion leaders—one senior nurse and one medical lead from each participating hospital	Staff in the Emergency Department responsible for the management of mTBI patients.	Local opinion leaders and staff in the Emergency Department responsible for the management of mTBI patients.
Setting	Participating hospitals	Participating hospitals	Off-site conference venue	Participating hospitals	Participating hospitals
Relevant BCTs for PTA behaviour	Information regarding behaviour, outcome.	Not applicable	Information regarding behaviour, outcome	Information regarding behaviour, outcome	Environmental changes
	Health consequences		Antecedents	Antecedents	Information regarding behaviour, outcome
	Persuasive communication		Health consequences	Health consequences	
	Social processes of encouragement, pressure, support		Goal/target specified behaviour or outcome	Graded task, starting with easy tasks	
			Graded task, starting with easy tasks	Increasing skills: problem solving, decision making, goal setting	
			Increasing skills: problem solving, decision making, goal setting	Modelling/demonstration of behaviour of others	
			Rehearsal of relevant skills	Persuasive communication	
			Modelling/demonstration of behaviour of others	Social processes of encouragement, pressure, support	
			Persuasive communication	Pros and Cons	
			Social processes of encouragement, pressure, support	Vicarious reinforcement	
			Pros and Cons	Social and environmental consequences	
			Vicarious reinforcement	Salience of consequences	
			Social and environmental consequences		
			Salience of consequences		
			Focus on past success		
Mode of delivery	Face-to-face meeting	One medical and nursing lead	Mixed, interactive and didactic workshop	Face to face workshops (mixed or clinician group specific depending on current training infrastructure in participating hospitals)	Printed copies
				Online presentations available for those not able to attend workshops	Online versions
					CT decision rules provided as lanyards
Intensity or dose	One meeting	Part-time	Two events in different Australian states	1 brief presentation per clinical topic, 1 demonstration session	For use with every patient
				Number of repeats left to LOLs	
Duration	One hour in length	Duration of the project	Full day	10–20 min per session	Not applicable

## Discussion

This paper illustrates a systematic, theory- and evidence-informed approach to developing an intervention that aims to improve the care of mTBI patients in the ED, that was informed by two theoretical frameworks: the TDF and the Model of Diffusion of Innovations in Service Organisations. Four evidence-based recommendations were identified to improve the care of this patient group, and the intervention components targeting the PTA behaviour consisted of 14 behaviour change techniques and addressed 6 TDF domains and 5 organisational domains. The modes of delivery were informed by six Cochrane reviews. There were five intervention components.

The TDF is frequently being used by researchers to explore clinical behaviour change and develop implementation interventions. It covers a range of behavioural influences including capability, motivation and opportunity; further elaboration of the domains to include organisation-level influences has been suggested [16]. It is recommended that studies targeting multiple levels (e.g. clinician and organisational) should draw upon multiple theories [62]. The benefit of studying change at the organisational level using organisational level theory, to complement the analyses regarding each recommended behaviour using the TDF, is that it facilitates exploration of the organisational context in greater detail and facilitates the inclusion of intervention components to directly target these influencing factors. There are limited practical examples in the literature of how to use theoretical frameworks when developing implementation interventions and this is, to our knowledge, the only study in the ED setting that has explicitly demonstrated how to use multiple theoretical frameworks to explore behaviour change and use these data to identify BCTs and develop intervention components.

The content of the intervention was designed to target hypothesised influences on behaviour and organisational change. This was achieved by selecting overarching strategies that were designed to address some of the organisational factors and/or maximise the likelihood that the intervention was fit for an organisational setting (e.g. stakeholder meetings and local opinion leaders), in addition to specifying BCTs relevant and tailored to each particular clinical behaviour. Synthesised evidence of professional behaviour change interventions and practical considerations of the mode of delivery informed development alongside theory and increased the likelihood that the end product was evidence-informed, feasible to deliver and acceptable to the ED community [63].

The core components of the intervention, the training of local opinion leaders to deliver local training workshops, addressed the majority of the identified facilitators of behaviour change using the TDF. The TDF domain ‘Environmental context and resources’ was not covered by the training components, and this domain was addressed with the provision of online and printed tools and materials, e.g. PTA assessment sheets and point of care reminder stickers. Intervention components, such as the involvement of senior leaders in local stakeholder meetings to create buy-in and the nomination of ‘multidisciplinary’ local opinion leaders to provide regular, brief training sessions in the ED, were chosen to target key organisational factors. There were, however, several intervention components that were deemed as not feasible for the ED setting. A major strength of this study, and the process used, is the documentation of decisions, throughout the process, of why intervention components were chosen and why they may have been modified. This enables researchers to understand the reasons for selecting content.

On conceptual grounds, there is reason to propose that the intervention, being based on robust theories and methods, is more likely to be effective than interventions that are not based on theory and evidence. However, it requires a cluster randomised controlled trial (cRCT) to address the empirical question as to whether this robust process leads to measurable effectiveness. The effectiveness of this intervention to improve care of patients with mTBI will be evaluated in a cluster randomised controlled trial [34] and outcome measures of behaviour change and factors thought to mediate the effect of the intervention along the proposed pathway of change will be assessed. These include mediators of behaviour change (e.g. beliefs about capabilities, beliefs about consequences), measures of practitioner behaviour (e.g. primary practitioner outcome is appropriate PTA screening), patient outcomes and cost. The evaluation of the factors along the causal pathway will be complemented by other components that form part of a process evaluation. The details of these outcomes and the process evaluation measures are reported separately [34]. Implementation research is a cumulative science, and this intervention is in the process of a robust evaluation that will add to the evidence of the effectiveness of theory-informed interventions to improve clinical practice.

Although there have been a number of publications on the development of theory-informed interventions to improve clinical practice [63–66], to our knowledge there have been few studies of this kind undertaken in the ED setting. A theory-informed intervention to implement two paediatric clinical pathways in the ED is being developed by Jabbour et al., but this is at the protocol stage [67]. Gould et al. are developing two theoretically enhanced audit and feedback interventions to improve the uptake of evidence-based transfusion practice using the TDF in combination with the Consolidated Framework for Implementation Research (CFIR) [64, 68]. The study is not focussed in the ED setting and is at the protocol stage. The research detailed in this paper may offer insights and guidance to those wanting to design implementation interventions in the ED setting and to those interested in using multiple theoretical frameworks, in addition to evidence and feasibility considerations in the design of implementation interventions.

One of the criticisms of past implementation research is the difficulty of understanding what intervention components were selected and their hypothesised mechanism of action [69]. This study followed a systematic process detailing how the intervention was developed and providing detailed descriptions of the intervention content. The intervention components have been described according to the WIDER and TIDieR Guidance [3, 4] and in terms of BCTs and modes of delivery [49, 66]. This differentiation between intervention content (BCTs) and models of delivery enables other researchers to explore the effectiveness of the BCTs when a different mode of delivery is applied [69].

The recent validation and refinement of the TDF domains has strengthened the rationale for its methodology and use in implementation research [15]. The validation of the TDF was published after the conduct of the interviews and therefore the original TDF was used to explore barriers and enablers with ED staff [14]. Although the BCTs were mapped to the original TDF domains, this process was supplemented with the BCTs proposed in the validation paper [48] linking the BCT Taxonomy v1 [49] to the refined TDF [15]. This taxonomy was recently updated to include 93 BCTs and 14 domains [66].

If theory is poorly operationalised, it will be less useful in identifying factors that influence outcomes in specified settings. Thus, an intervention may be ineffective due to the research team’s operationalisation of theory when developing the intervention [8]. This is potentially a methodological limitation of this study; although we used a systematic and replicable process to operationalise the theoretical domains in terms of appropriate intervention components, the process was conducted by just one research team. There is, however, little research on how best to operationalise theory in the context of intervention development and selecting or designing intervention components [70]. The research team did, however, include a wide range of ED clinicians, behavioural scientists and evidence-based researchers to incorporate a breadth of experience.

## Conclusions

This paper provides a systematic, theory- and evidence-informed approach to developing an intervention aiming to change professional practice in the ED setting. Theoretical frameworks, evidence-based behaviour change techniques, evidence about the effects of modes of delivery (EPOC systematic reviews) and feasibility information were systematically brought together to develop an intervention that aims to improve the management of mTBI patients in the ED. This study demonstrated the use of the TDF in addition to a model designed to explore organisational factors to develop a theory-informed intervention in a complex organisational setting. The effectiveness of this intervention will be evaluated in a large national cluster randomised controlled trial which forms part of a larger program of work called the Neurotrauma Evidence Translation (NET) program [33, 34].

## Abbreviations

mTBI: Mild traumatic brain injury
ED: Emergency department
TDF: Theoretical domains framework
PTA: Post-traumatic amnesia
NET: Neurotrauma evidence translation
BCT: Behaviour change techniques
EBG: Evidence-based guideline
EPOC: Effective practice and organisation of care
